# Cloning and expression of an anti-cancerous cytokine: human *IL-29* gene in *Chlamydomonas reinhardtii*

**DOI:** 10.1186/s13568-023-01530-1

**Published:** 2023-02-25

**Authors:** Maham Akram, Mohsin Ahmad Khan, Nadeem Ahmed, Rashid Bhatti, Rabbia Pervaiz, Kausar Malik, Saad Tahir, Rabia Abbas, Fareeha Ashraf, Qurban Ali

**Affiliations:** 1 Centre for Excellence in Molecular Biology (CEMB), University of the Punjab, 87-West Canal Bank Road Thokar Niaz Baig, Lahore, Pakistan; 2 Department of Plant Breeding and Genetics, Faculty of Agricultural Sciences, University of the Punjab, Lahore, Pakistan

**Keywords:** Interleukin 29 (*IL29*), Therapeutic protein, Chloroplast expression, *Chlamydomonas reinhardtii*, TN72 strain

## Abstract

Green algae, *Chlamydomonas reinhardtii*, with low cultivation cost, absence of endotoxins and insusceptibility to human pathogens is emerging as a potential system for the future production of recombinant proteins. The recent development of molecular tools enabling recombinant protein expression in algae chloroplast has provided new research and advance opportunities for developing low-cost therapeutic proteins. In the present study, algae chloroplast expression system was evaluated for the recombinant production of an anti-cancerous therapeutic protein, Interleukin 29 (*IL29*). The *IL29* gene was cloned into algae chloroplast expression vector (pSRSapI). After the transformation, the positive clones were screened for homoplasmy and the presence of the *IL29* gene by spot test and PCR analysis, respectively. The expressed *SDS-PAGE and western blotting assay characterized IL-29*. The algae expressed *IL-29* was biologically active in an anti-proliferating bioassay using HepG2 cells. The results suggest that the *Chlamydomonas reinhardtii* expression system is convenient, low-cost, eco-friendly, and safe to express *IL29*.

## Key points

- The *IL29* gene cloning in pSRSapI.
- *IL-29* found as biologically active.

## Introduction

The world of science and industry has been revolutionized with recombinant protein production. Microbes are the major hub to produce the majority of recombinant proteins. *Escherichia coli* (bacteria), *Pichia pastoris* and *Saccharomyces cerevisiae* (yeast), insects, transgenic animals and plants, and mammalian cell lines are used mainly for their production (Shamriz and Ofoghi 2016). Every expression system comprises various benefits in terms of safety, convenience in manipulation and yield, proper folding of proteins and production cost. In past decades, molecular farming has become a source of attention as an appropriate production platform for various therapeutic proteins. Molecular farming offers various advantages, including low production cost, pathogen and toxin-free protein production, ease of culturing, harvesting and purification, and high quality and bioactive protein (proper protein folding and glycosylation) (Ganapathy 2016; Gecchele et al. 2015; Shamriz and Ofoghi 2016). Despite having some advantages, it possesses some disadvantages like production takes much time as it depends on plant growth (Giritch et al. 2006), gene flow from crop to crop is another major concern (Ellstrand 2001), the downstream processing is much more expensive, and in most cases, it reduces quality and yield of recombinant protein (Kong et al. 2001). In such a situation, microalgae is a quite appealing substitute for molecular farming for producing therapeutic proteins (Mathieu-Rivet et al. 2014).

Microalgae belong to a polyphyletic group of photosynthetic eukaryotic organisms that can survive in photosynthetic, heterotrophic and mixotrophic regimes in terrestrial and aquatic environments (Scaife et al. 2015). Microalgae have become a great source of interest in producing various recombinant proteins and metabolites. Various molecular biology tools have been designed for proficient genetic engineering and microalgae system modifications in the recent era. Progress in this field has been made by using *Chlamydomonas reinhardtii* as a model organism specifically because of its simple reproduction cycle, culturing conveniences and well-known genetics (Shamriz and Ofoghi 2016).

A unicellular, biflagellate green algae *Chlamydomonas reinhardtii* found in freshwater almost worldwide (Khan et al. 2020). The *C. reinhardtii* system is being used as a model organism because of its various significant attributes; (1) Short reproduction, cultivation and harvesting time and scale-up production period of recombinant proteins is short as compared to transgenic plants and animals (Mayfield et al. 2007), (2) Post-translational modifications, (3) Ease of transformation (mitochondrial, nuclear and chloroplast), (4) It can grow under both phototrophic and heterotrophic habitat in outdoors and bioreactors respectively, (5) For the genetic modifications of the cell, a vast range of promoters and markers are accessible and (as GRAS by FDA so that it can be administered orally (in case of vaccines) or as an enriched feed (Purton et al. 2013; Shamriz and Ofoghi 2016). The nuclear and chloroplast transformation are mostly used for genetic manipulation. Still, chloroplast transformation is more widely used as compared to nuclear because of its disadvantages, such as random gene integration, silencing of transgene, and epigenetic and positional effects which eventually causes low yield of recombinant protein (Rasala and Mayfield 2011; Specht 2014; Doron et al. 2016). Comparatively, site-directed gene integration (homologous recombination), disulfide bond formation, no chance of gene silencing and robust expression are the advantages of chloroplast transformation. The only limitation is that it does not provide desirable glycosylation, making it best for producing proteins with minimal or no glycosylation (Almaraz-Delgado et al. 2014; León et al. 2008; Mayfield et al. 2007). Furthermore, the possibilities of proteolytic cleavage are quite low, and the chloroplast attains 40% of the total cell mass, making genetic engineering easier (Mayfield et al. 2007).

Human interleukin-29 (IL29), also known as IFNλ1 has antiproliferative, immunomodulatory and antiviral effects that play an important role in adaptive and innate immunity. IL29 genes induce the production of MHC class I molecules that help to present antigens on the cellular surface of infected cells, which is an essential part of adaptive immunity. *IL29* was expressed previously in the expression system of *E. coli* and Iranian Lizard *Leishmania*. All of these systems are cost-ineffective, and purification steps are complicated except for *E. coli*, which has the drawback that it lacks post-translational modifications. So, the present study was carried out for the first time to evaluate the potential of *Chlamydomonas reinhardtii* to produce *IL29*. A cell wall deficient wild-type strain cc-5168 or TN72 (cw15, *∆psbH*, SpecR) of *Chlamydomonas reinhardtii* has been used in the current study. This wild-type strain is PSII-deficient (i.e. acetate-dependent) and spectinomycin-resistant (due to the presence of *aadA* cassette). TN72 is used as a recipient strain for chloroplast transformation with the Purton lab's pSRSapI expression vector. After expression and protein extraction, the anticancer potential of purified *IL29* was determined utilizing a cell viability assay. Production of biologically active protein (*IL29*) demonstrates that algal chloroplast can be used to produce recombinant human interleukin-29 for therapeutic purposes.

## Materials and methods

### Culture conditions for *Chlamydomonas reinhardtii*

The chloroplast transformation was done using the cell wall deficient strain of *Chlamydomonas reinhardtii* named CC-5168 (TN72) obtained from Chlamydomonas Resource Centre (http://www.chlamycollection.org). Spectinomycin was used for the selection of the strain. The algal strains were maintained and grown on Tris–acetate phosphate medium (TAP) (1 M Tris, TAP salts, phosphate buffer, acetic acid, hunter trace elements and d.H_2_O) using 30 μmol photons m^–2^ s^–1^ light intensity and 25 °C temperature. High salt minimal (HSM) medium (Beijernick salts, phosphate buffer, hunter trace elements and d.H_2_O) was used for transformation and homoplasmy achievement. The broth cultures were grown with continuous shaking at 100 rpm for 4–5 days at 25 °C in a continuous light period having 30 μmol photons m^–2^ s^–1^ light intensity (Charoonnart et al. 2019; Braun‐Galleani et al. 2015).

### Plasmid construction

The gene sequence of the human IL29 (IFNλ1) was obtained from NCBI (Gene ID: 282618). Restriction sites *Sap*I and *Sph*I were added in the 5′ and 3′ ends of the gene sequence to facilitate cloning in the expression vector of *Chlamydomonas reinhardtii* pSRSapI. The gene sequence with added restriction sites was provided to the gene synthesis company (Molecular products and Co. MPC, Pakistan), which provided us with the synthesized gene (*IL29)* in pet28. The gene was amplified using *IL29* specified primers (forward primer: 5′-GCT CTT CAA TGG GTC CGG TG-3′ and reverse primer 5′-GCA TGC TTT AGG TAG ATT CCG GGT G-3′). The amplified PCR product was purified using a quick gel extraction kit (Invitrogen Cat no: K210025), digested with *Sap*I and *Sph*I enzymes and then ligated into a linearized pSRSapI vector which was also digested through *Sap*I and *Sph*I enzymes. The detailed structure of pSRSapI expression vector was previously described (Wannathong et al. 2016). The algal transgenic lines of *Chlamydomonas reinhardtii* were selected on ampicillin based on the presence of a *psbH* marker.

### Transformation of *Chlamydomonas reinhardtii*

The transformation of wild-type *C. reinhardtii* (cc5168) was done using the previously described method (Kindle et al. 1991). A 200 mL culture of wild-type cc5168 was used for chloroplast transformation, which was grown to the early log phase (2 × 10^6^ cells/mL). The cells were concentrated by centrifugation, and the final pellet was suspended in a 2 mL TAP medium. In a reaction tube, 300 μL cells, 300 mg sterile glass beads (400–625 nm diameter) and 5–10 μg plasmid DNA were agitated at high speed for 15 s on vortex. After agitation, 500 μL 0.5% molten HSM agar (42 °C) was added, mixed and spread on 1.5% HSM agar plates. The plates were incubated in the dark (~ 2 μE/m^2^/s light intensity) overnight at 25 °C and then on moderate light (~ 50 μE/m^2^/s light intensity) after 24 h. Transformed colonies were obtained after 2–3 weeks and repeatedly restreaked to obtain a pure transformant line. Homoplasmicity was checked through spot test and PCR using specific primers (Table 1). The genomic DNA from the algal strains was isolated using Scott Newman’s protocol (Newman et al. 1990), and PCR was done with homoplasmy checking specific primers and gene-specific primers.

**Table 1:** Primers used for confirmation of homoplasmy

Primers	Sequences
F1	GTCATTGCGAAAATACTGGTGC
R1	CGGATGTAACTCAATCGGTAG
R2	ACGTCCACAGGCGTCGTAAGC

### Protein extraction

Protein from the *C. reinhardtii* transformants and wt. cc5168 was extracted from a 100 mL culture which was allowed to manipulate at 25 °C for 3–4 days under constant moderate light (~ 50 μE/m^2^/s) up to the mid-log phase (2 × 10^6^ cells/mL). 1 mL culture was piped out in a spectrophotometer cuvette, and cell density was measured at 750 nm using a spectrophotometer. 10 mL culture was centrifuged in a 20 mL centrifuge tube at 5000 rpm for 5 min, after which the supernatant was completely aspirant off. Solution A (0.2 M Sorbitol, 0.8 M Tris–Cl, and 1% beta-mercaptoethanol), Y/2 mL, was used for resuspension, where Y stands for optical density measured at 750 nm (Braun Galleani 2014). This ensures that all samples are at the same concentration. Protein was transferred into 1.5 mL microcentrifuge tubes and stored immediately at − 20 °C for short-term use.

### Protein quantification

The total soluble protein extracted from the samples was quantified using the standard Bradford assay protocol. Quick Start™ Bradford Protein Assay Instruction Manual was followed for microplate assay. The protein from transformants and wild-type strains was quantified.

### SDS-PAGE and western blot analysis of extracted protein

Fifty microliter sample was mixed with 5.5 μL 10%SDS, boiled for 5 min at 99 °C and centrifuged at 15,000*g* for 5 min. The supernatant was used for SDS-PAGE analysis (Braun Galleani 2014). Protein samples were fractioned on 12% acrylamide SDS-PAGE gel and then blotted onto a nitrocellulose membrane. For immunodetection, membrane blocking was done by overnight incubation at 4 °C in 0.5% skim milk powder dissolved in TBS-T (20 mM Tris, 137 mM NaCl, 1 M HCl pH 7.4, 0.1% Tween-20). After blocking, the membrane was incubated for 1 h at room temperature (RT) in 1:5000 diluted IL-28/29 mouse monoclonal antibody (H-1) (Santa Cruz sc-365834), dissolved in 0.5% skim milk powder in 1X TBS-T, washed 3 times for 5 min in 1X TBS-T and then incubated in 1:10,000 diluted goat anti-mouse IgG-AP secondary antibody (Santa Cruz sc-2008) in 1X TBS-T for 1 h at room temperature. Washing was done again the same as before, and the IL29 gene was visualized by adding NBT/BCIP, tablet solution (1 tablet in 10 mL distilled water) (Young and Purton 2014).

### Cell viability assay

MTT assay was performed to investigate the anticancer activity of recombinant IL29 total soluble protein. Briefly, 2 × 10^4^ HepG2 cells were seeded in a 96-well plate, and cultured in high glucose DMEM supplemented with 10% FBS and 1% penicillin–streptomycin at 5% CO_2_ and 37 °C. After monolayer formation, the medium was refreshed, and the cells were treated with purified recombinant IL29 at concentrations of 100 μg/mL and 200 μg/mL. After 24 h, the old media was discarded, and the cells were incubated with 100 μL medium and 20 μL MTT solution (5 mg/mL) for 3 h at 37 °C in 5% CO_2_. Later, the media was discarded, and 100 μL of DMSO was added to each well to dissolve the formazan crystals. The experiment was carried out in triplicates, and absorbance was measured at test wavelength 570 nm and reference wavelength 620 nm using a microplate reader (BMG Labtech, Germany).

### Statistical analysis

MTT assay was performed in technical triplicates (n = 3), and one-way ANOVA was performed with a significance level of p ≤ 0.05. GraphPad prism v.6.00 (GraphPad Software, San Diego, California, USA) was used for statistical analysis. The data were expressed as the standard deviation of the mean.

## Results

### Construction of plasmid with *IL29* gene

**Fig. 1:**
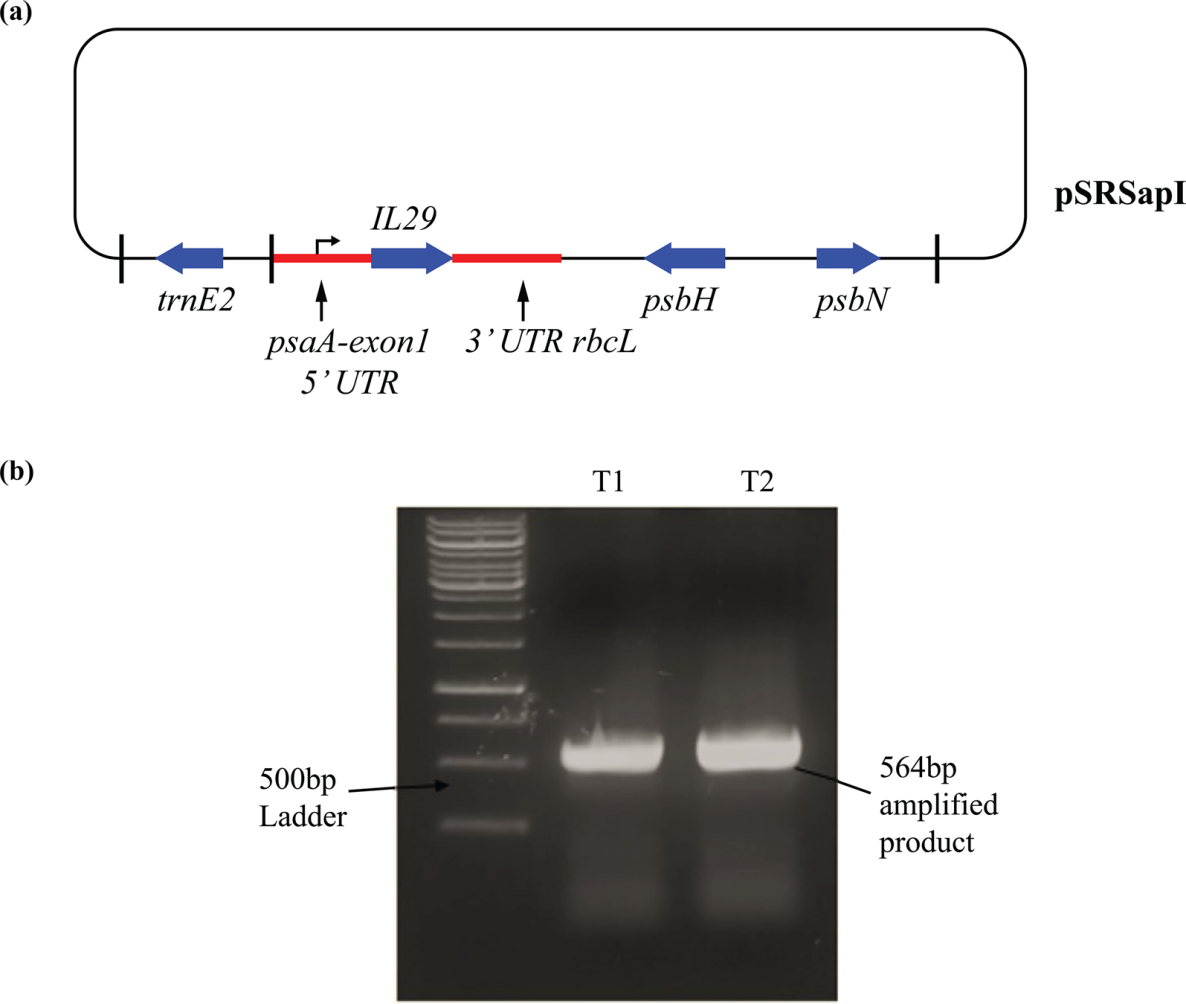
Generation and confirmation of *IL29* transformants. **a** The pSRSapI-*IL29* plasmid contains *IL29* under the control of the *psaA* exon-1 promoter/5′UTR and *rbcL* 3′UTR. This construct comprises *psbH* mutated in the TN72 strain of *Chlamydomonas reinhardtii.* **b** PCR confirmation of *IL29* integration in pSRSapI expression vector through gene-specific primers. Transformants yield the 564 bp product

*IL29* gene was ligated into pSRSapI expression vector, and recombinant plasmid pSRSapI-*IL29* (Fig. 1a) was transformed into *E. coli* TOP10’ strain. After selecting recombinant strains, recombinant plasmid DNA was isolated from bacterial cells, and PCR was performed using the gene-specific primers that showed the presence of *IL29* (Fig. 1b).

### Transformation of the recombinant plasmid into *C. reinhardtii*

**Fig. 2:**
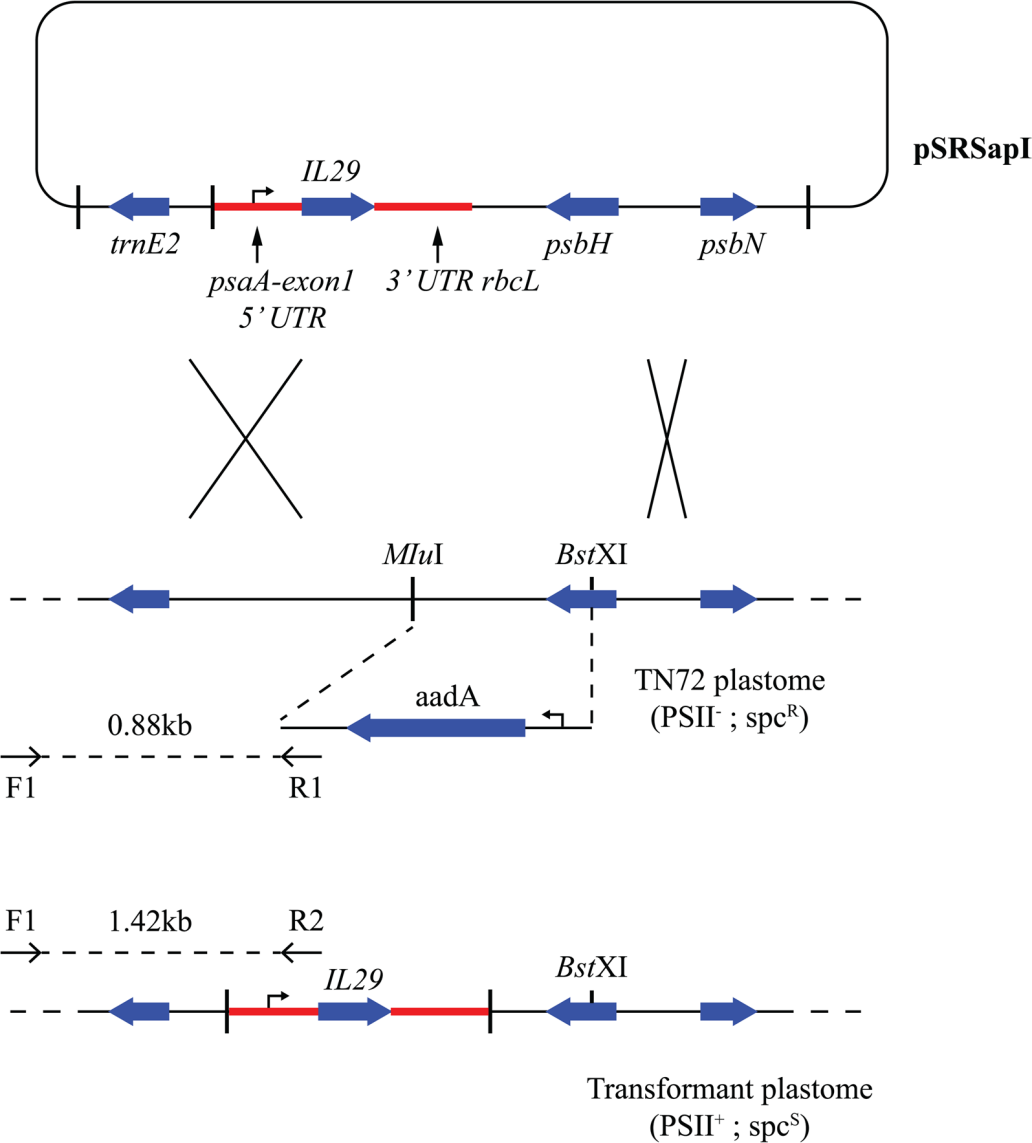
Mechanism of homoplasmy; Integration of *IL29* and loss of *aadA* cassette in plastome of TN72 occurs by two homologous recombination events

**Fig. 3:**
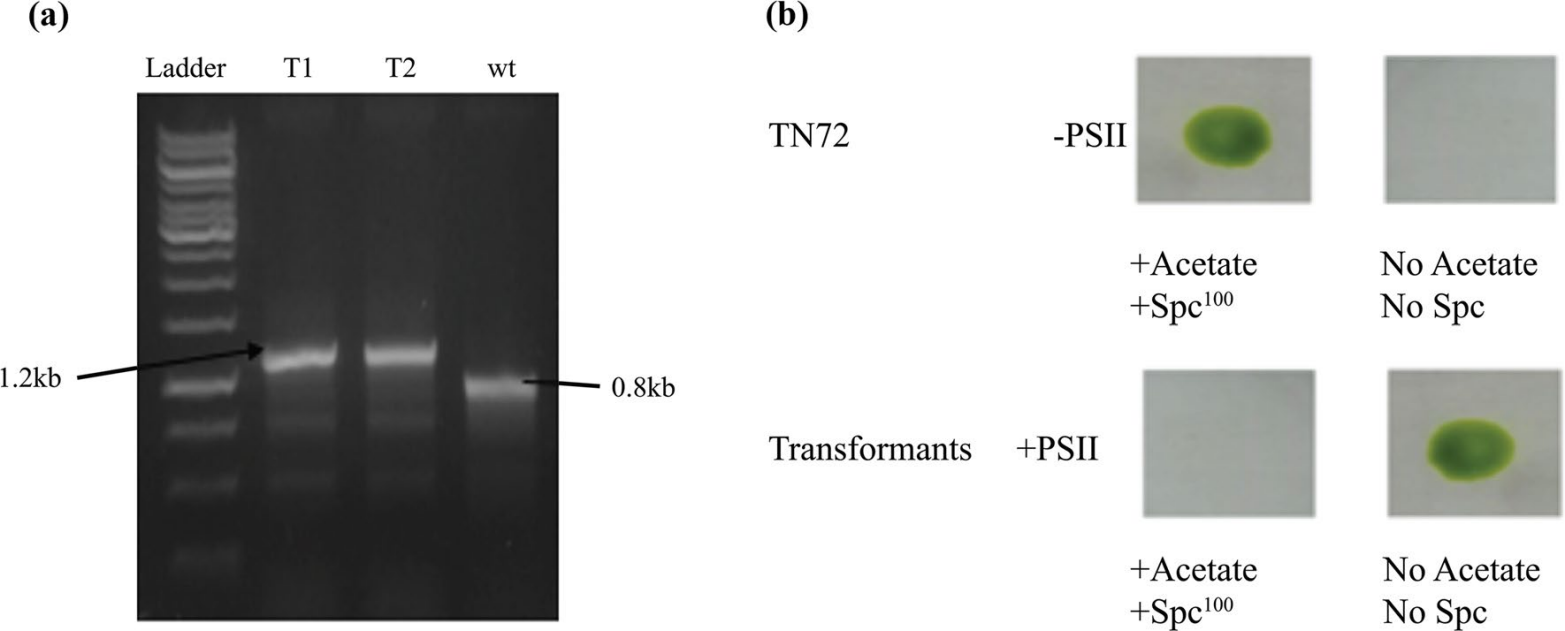
Confirmation of homoplasmy by PCR and spot test. **a** The original strain of *Chlamydomonas reinhardtii* TN72 gives a product of 0.88 kb with F1 and R1 primers, while the transformants yield a product of 1.2 kb with some non-specific amplifications. The presence of a 1.2 kb band instead of a 0.88 kb band confirms that all transformants are homoplasmic. **b** Spot test shows that the original strain of TN72 grows in the presence of spectinomycin (spec) as it consists of spec resistant cassette (upper panel), which is lost after transformation due to which homoplasmic transformants cannot manifest phototrophic growth in the presence of spectinomycin (lower panel). + PSII; the presence of photosystem II, -PSII; absence of photosystem II

The recombinant construct pSRSapI-*IL29* was transformed into a cell wall deficient strain of *Chlamydomonas reinhardtii* TN72 (cc5168). Transformation of the algal cells was done by the agitation method. Transformants were allowed to grow on HSM agar plates for 2–3 weeks and further streaked to get the homoplasmic lines (Fig. 2). Furthermore, homoplasmy was confirmed through PCR using homoplasmy-specific primers F1 and R1 (Fig. 3a). Spot test was also performed on the selected transformants T1 and T2 to confirm the homoplasmy (Fig. 3b).

### Protein analysis through SDS-PAGE and western blotting

**Fig. 4:**
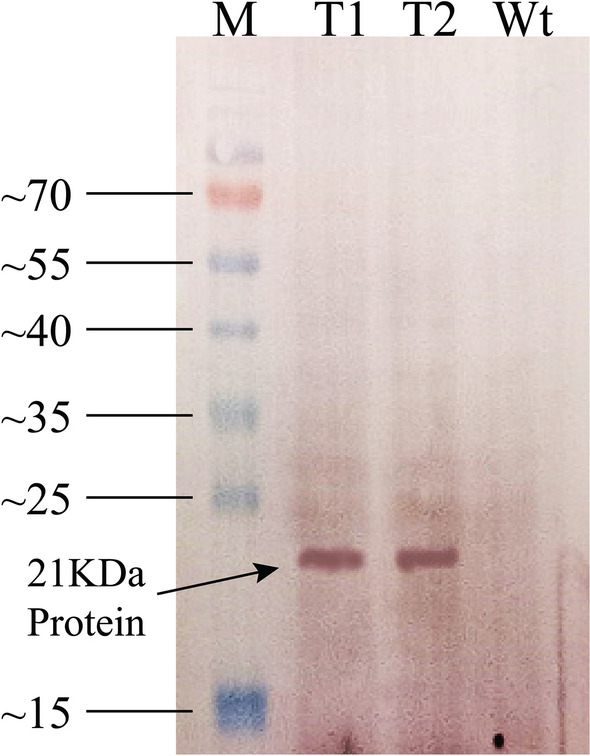
Western blot analysis of extracted crude protein. *Lane1*: Thermofisher prestained protein ladder (cat no: 26617)*. Lane 2 and lane 3:* protein samples T1 and T2*. Lane 4:* wild type TN72

The expression of *IL29* in algal cells was studied through protein analysis. Protein was isolated from the algal cells, quantified through Bradford assay, and SDS-PAGE was run to check the expression of a protein. The protein quantification showed 2.79 mg/mL of TSP of T1 and T2 and 2.18 mg/mL of wild-type TSP. The study was done through both Coomassie Blue staining and silver staining. Two strains labeled T1 and T2 were selected for protein analysis. TN72 (wilt type) was run as a control for the evaluation. The protein extracted and analyzed on SDS-PAGE was in crude form. 25 µL and 30 µL of T1 and T2 were loaded along with 25 µL of TN72. The result showed the presence of a 21 kDa protein band. Extracted crude protein was confirmed with western blot analysis using a gene-specific primary antibody and AP (Alkaline phosphatase) conjugated secondary antibody. The result showed the presence of a specific protein in the sample (Fig. 4).

### Inhibition of HepG2 cells growth by IL29

**Fig. 5:**
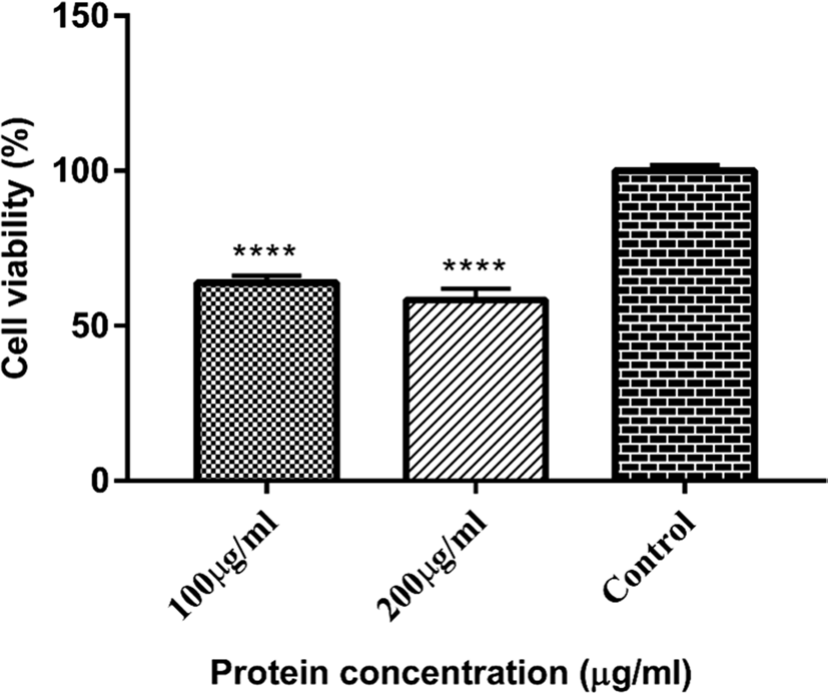
Percentage viability of HepG2 cells at 100 µg/mL and 200 µg/mL dose concentrations of purified recombinant *IL29* protein

To evaluate the effect of IL29 on HepG2 cells, cell survival was detected by MTT assay. HepG2 cells were treated with 100 µg/mL and 200 µg/mL for 24 h. As shown in Fig. 5, IL29 strongly inhibited liver cancer cells growth in a dose-dependent manner from 100 to 200 µg/mL dose concentrations. Although both doses showed growth inhibition, significant inhibition was observed at 200 μg/mL.

## Discussions

Plant molecular farming is being used to synthesize various biopharmaceuticals, industrial proteins and bioactive metabolites (Murphy and chemistry 2012). Green plants and microalgae could be transformed into bio-factories to synthesize recombinant proteins (Griesbeck and Kirchmayr 2012; Obembe et al. 2011). Microalgae-based systems can combine the favorable characteristics of microorganisms and plants, and they have emerged as a viable option for molecular farming. Compared to typical molecular farming systems, *Chlamydomonas reinhardtii* offers numerous benefits, including affordable cultivation, improved biosafety, and the capacity to fold proteins precisely (Rosales-Mendoza et al. 2012). The current success in producing numerous recombinant therapeutics in *Chlamydomonas reinhardtii* chloroplast has opened the door to the future use of this alga as a commercial factory (Wannathong et al. 2016). Interleukin 29 (*IL-29*) was discovered in 2003 and belonged to the type II cytokine subfamily. In humans, it is the most potent and abundant interferon molecule in serum (Hamming et al. 2010). Multiple studies have also reported that *IL-29* plays a role in cancer etiology and has anticancer properties (Kelm et al. 2016).

*Chlamydomonas reinhardtii* has proven to be an effective platform for recombinant protein/peptide production (Reyes-Barrera et al. 2021; Li et al. 2021; Jiang et al. 2021). In this study, the *IL29* protein was first expressed in an algal expression system. Previously, bacteria was used for its production (Li and He 2006). The bacterial expression system is the first choice for expressing proteins and several peptides because of its convenience in growth, elevated rate of production, and proficiency in genetic modification. On the contrary, they have some limitations, like they are incapable of carrying out protein modifications, which are compulsory for stability and proper protein functioning, especially in the case of human proteins. Moreover, these proteins are produced as insoluble inclusion bodies that need refolding and purification, which is costly and inadequate. Another drawback is that several endotoxins are produced by a bacterial system, which can be the reason for complications in purification and the appliance of these recombinant proteins (Gao and Tsan 2003).

A cell wall deficient wild-type strain cc-5168 or TN72 (cw15, ∆*psbH,* SpecR) (http://www.chlamycollection.org) of *Chlamydomonas reinhardtii* and pSRSapI (recipient vector) used in this study provides a simple and low-cost system for developing a transgenic line within a short time. The choice for chloroplast transformation over nuclear was made because of random insertion of transgene, due to which they vary in their expression level from protein to protein; also, a foreign gene can be silenced because of transcriptional processes, e.g., methylation of cytosine and posttranscriptional processes (Schroda 2006; Cerutti et al. 1997). While in the chloroplast, site-specific recombination takes place, protein expression does not vary, and silencing is not observed (Debuchy et al. 1989). The transgenic lines were obtained by transforming TN72 through the agitation method (using glass beads) instead of the electroporation method. Transformants were selected based on their sensitivity to spectinomycin. As described previously, TN72 is devoid of functional photosystem II (PSII) as its main gene *psbH* is knocked out. Instead of this, another gene *aadA* is present, which works as a selection marker as it makes this wild type strain resistant to spectinomycin. After the transformation with the pSRSapI vector (keeping *IL29* and *psbH* gene), homologous recombination occurs with the chloroplast genome, and *aadA* gene is replaced with the *psbH* gene and *IL29* gene. This restores PSII activity, and transformants become susceptible to spectinomycin. After achieving transgenic lines, the protein was extracted, quantified, and analyzed through western blotting. According to the results, as shown in Fig. 4, recombinant protein *IL29* was well expressed in the plastome of *Chlamydomonas reinhardtii* with the reported molecular weight (Kotenko et al. 2003).

The IL29 protein yield was 0.61 mg/mL, which is in crude form, and much more than expressed in Iranian Lizard *Leishmania* 0.075 mg/L (Taromchi et al. 2013) and *E. coli,* which is 60 mg/L (Li and He 2006). Modifications in purification steps and the addition of tags can help in better yield.

The MTT assay was used to determine the in vitro cytotoxicity of the recombinant *IL29* on the proliferation of the HepG2 cell line. Two doses of protein extract, 100 µg/mL and 200 µg/mL were tested. Both doses showed a significant cytotoxic effect on the HepG2 cell line. HepG2 cell line has been widely used to assess the anti-tumor activities of various proteins and plant extracts (El-Garhy et al. 2017). The antitumor effects of cyanobacterial l-asparaginase produced in *E. coli* (Kebeish et al. 2016) or isolated from *Helicobacter pylori* (Gladilina et al. 2009) and *Penicillium brevicompactum* (Elshafei et al. 2012) were assessed by using the HepG2 cell line. Furthermore, several forms of human interferon have been classified based on their inhibitory effects on Hep3B and HepG2 cell growth (Zhou et al. 2007). This study suggests that *IL29* expressed in *C. reinhardtii* has significant anticancer effects.

Algae offer a prospective platform for the large-scale fabrication of a wide range of recombinant proteins due to the exponential growth rate and the lack of general pathogens with *Homo sapi*ens. The foremost benefit that algae offer is the correspondence between the mechanism of human and algae protein synthesis. More improvements in this work are needed, like adding a tag for immunoprecipitation can facilitate the extraction of purified protein. Furthermore, despite some lacking IL29 expressing alga can be used as a dietary supplement as alga is regarded as a GRAS organism. This advantage of alga makes it superior to other expression systems.

Microalgae are simple to grow and process, making them a more cost-effective platform for therapeutic protein production. *C. reinhardtii*, green algae, has proven to be an effective biopharmaceutical expression platform. For the first time, an *IL29* expression cassette was developed and expressed in *C. reinhardtii*, yielding a bioactive protein with anticancer activity. These encouraging results suggest that the IL29 may be produced at a reasonable cost. Thus, the success rate and production of therapeutically active IL29 demonstrate *Chlamydomonas reinhardtii's* value as a cell factory for producing human therapeutic active proteins.

## Data Availability

All data generated or analyzed during this study are included in this published article.

## Abbreviations

*C. reinhardtii*: *Chlamydomonas reinhardtii*
*E.coli*: *Escherchia coli*
TAP: Tris acetate phosphate
HSM: High salt media
GRAS: Generally recognized as safe
FDA: Food and Drug Administration
Interleukin-29: IL29/IFN-λ1
MHC: Major histocompatibility complex
PCR: Polymerase chain reaction
SDS-PAGE: Sodium-dodecyl polyacrylamide gel electrophoresis
TSP: Total soluble protein
NaCl: Sodium chloride
HCL: Hydrochloric acid
TBS-T: Tris buffer saline-Tween20
IgG-AP: Immunoglobin G-alkaline phosphatase
NBT/BCIP: Nitro blue tetrazolium/bromo-4-chloro-3-indolyl phosphate
DMEM: Dulbecco’s Modified Eagle Medium
FBS: Fetal bovine serum
DMSO: Dimethyl sulfoxide
MTT: 3-(4,5-Dimethylthiazol-2-yl)-2,5-diphenyltetrazolium bromide
HepG2: Human liver cancer cell line
